# Socioeconomic determinants of sarcopenic obesity and frail obesity in community-dwelling older adults: The Seniors-ENRICA Study

**DOI:** 10.1038/s41598-018-28982-x

**Published:** 2018-07-17

**Authors:** Belén Moreno-Franco, Raúl F. Pérez-Tasigchana, Esther Lopez-Garcia, Martin Laclaustra, Juan L. Gutierrez-Fisac, Fernando Rodríguez-Artalejo, Pilar Guallar-Castillón

**Affiliations:** 1 Translational Research Unit, Hospital Universitario Miguel Servet, Instituto de Investigación Sanitaria Aragón (IIS Aragón), Universidad de Zaragoza, CIBERCV, Zaragoza, Spain; 2 Department of Preventive Medicine and Public Health, School of Medicine, Universidad Autónoma de Madrid/IdiPaz, CIBERESP, Madrid, Spain; 3 School of Medicine, Universidad San Francisco de Quito, Quito, Ecuador; 4 IMDEA-Food Institute, CEI UAM+CSIC, Madrid, Spain

## Abstract

Information on the association between socioeconomic status (SES) throughout life and sarcopenic obesity is scarce, whereas no study has been focused on the association between SES and frail obesity. This analysis estimated the prevalence of sarcopenic obesity and frail obesity, and their associations with SES in older adults. Data were collected in 2012 from 1,765 non-institutionalized individuals aged ≥65 participating in the Seniors-ENRICA study in Spain, by using standardized techniques and equipment. SES throughout life was evaluated with the father’s occupation, participant’s educational level, former own occupation, and current poor housing condition. Overall, 17.2% of participants had sarcopenic obesity, and 4.0% frail obesity. No association was found between SES and sarcopenic obesity. In contrast, the prevalence of frail obesity was higher in those with lower education, having worked in manual job, and currently having poor housing condition. Having ≥1 social disadvantages throughout life was associated with higher prevalence of frail obesity. The prevalence of this disorder increased by 1.49 (95% CI: 1.21–1.85) times for each social disadvantage added. The OR (95% CI) of frail obesity was 3.13 (1.71–5.7) for those having 3 or 4 vs. 0 or 1 social disadvantages, implying a more complex process beginning early in life.

## Introduction

The aging process is associated with loss of skeletal muscle mass (SMM) and strength, a phenomenon known as sarcopenia, which in turn is linked to higher risk of functional impairment and death^1^. Frailty is a geriatric syndrome characterized by loss of physiological and functional reserve that increases the vulnerability to even minor stressors (e.g.: dehydration, a cold, diarrhea, etc.)^2^. As a result, frailty is associated with greater risk of falls, hospitalization, disability, and death^3^. Sarcopenia can be in the pathophysiological pathway to frailty, but they are independent syndromes.

Simultaneously with the process of sarcopenia that occurs in the elderly, there is an increase in fat mass, that is reflected in the high prevalence of obesity in recent decades worldwide^4^. Obesity induces a pro-inflammatory state through the release of adipokines such as IL-6 and TNF-α which can increase muscle loss, and also modify muscle composition and quality, potentially affecting its functionality^5–7^. Thus, despite a low body mass index (BMI) it is still a predictor of sarcopenia^8^, also excess of adiposity couples with sarcopenia, characterizing the so called “sarcopenic obesity”. It has been postulated that the synergistic association between sarcopenia and obesity may potentiate the effects of both syndromes separately, playing a probable role in the increased risk of cardiovascular disease and even of mortality. Nevertheless the results are still contradictory and dependent on the heterogeneity of the definitions of these syndromes^9,10^. In addition, although frailty is considered a wasting disorder, it can coexist with obesity as “frail obesity”. In fact, so far today, longitudinal studies have only considered obesity as a determinant of frailty^7,11,12^, without considering frail obesity as a proper entity. Previously reported prevalence of sarcopenia and sarcopenic obesity in the Spanish population ranged between 13.8 and 24.1%, and 11.0 and 14.9% respectively^13,14^. In addition, prevalence of frailty found in Spanish studies spans from 8.4 to 16.9, which makes it a potential problem for the aging Spanish demographics^15–17^.

Inequalities in socioeconomic status (SES) throughout life are associated with increased risk of morbidity and mortality^18–22^, and can lead to poor health outcomes later in life. It is well known that SES influences obesity^23^. Likewise, its relationship with sarcopenia and frailty has previously been studied^24,25^. However, information on the association between SES throughout life and sarcopenic obesity is scarce^26^, whereas no study has been focused on the association between SES and frail obesity. So, this study assessed the prevalence of sarcopenic and frail obesity, and its association with SES throughout life, in community-dwelling older adults.

## Methods

### Study design and participants

We used cross-sectional data from the second wave of the Seniors-ENRICA cohort^7,27^. Briefly, this cohort was set up in 2008–2010 with 3,289 individuals, representative of the non-institutionalized Spanish population aged ≥60^27^. Participants were followed up to 2012, when a second wave of data collection was performed. Data in this second wave were collected during a telephone interview on socio-demographic variables, lifestyle and morbidity. There were two subsequent home visits to obtain blood and urine samples, perform a physical examination, record habitual diet, and prescribed medication. After excluding 95 participants who died, 2,519 individuals provided updated information; of them, we excluded 264 participants aged < 65, and 490 with missing data on anthropometry, impedanciometry, or SES components. Thus, the analyses were conducted with 1,765 subjects.

The Seniors-ENRICA study was approved by the Clinical Research Ethics Committee of *La Paz* University Hospital. All research was performed in accordance with relevant guidelines, and study participants gave informed written consent.

### Study variables

Weight, height and percentage of body fat (%BF) were measured in all participants. Height was measured twice with a portable extendable stadiometers (model Ka We 44 444 Seca). Weight and %BF was estimated by bioelectrical impedance analysis (BIA) (Tanita®SC-240MA, Tanita Corp., Tokyo, Japan). BMI was calculated as weight in kg divided by height in meters squared.

SMM (kg) was calculated with the equation developed by Janssen *et al*.^28^: ([Height^2^/Resistance − 0.401] + [sex × 3.825] + [age × −0.071]) + 5.102, where height is given in cm, resistance in ohms (from BIA), sex as 1 for men and 0 for women, and age in years. Skeletal muscle mass index (SMI) was estimated by dividing SMM by height in meters squared. Sex-specific quintiles of SMI were created: ≤9.21; 9.22–10.06; 10.07–10.75; 10.76–11.67; ≥11.69 for men, and ≤7.07; 7.08–7.70; 7.71–8.31; 8.32–9.18; ≥9.19 for women. Sarcopenia was defined as the lower two quintiles of SMI^29^. Sex-specific quintiles of %BF were: ≤22.30; 22.40–25.40; 25.44–28.70; 28.80–32.60; ≥32.68 for men, and ≤31.80; 31.82–35.70; 35.80–39.10; 39.20–42.60; ≥42.70 for women. Participants were classified as obese, when they were in the upper two quintiles of %BF.

Frailty was defined as having at least three of the five Fried’s criteria^30^: (1) exhaustion: any of the following responses to two questions taken from the Center for Epidemiologic Studies Depression Scale^31^: “I feel that anything I do is a big effort” or “I feel that I cannot keep on doing things” at least 3–4 days a week; (2) weight loss: unintentional loss of ≥4.5 kg of body weight in the preceding year; (3) low physical activity: walking ≤2.5 h/week in men or ≤2 h/week in women^31^; (4) weakness: the cohort-specific lowest quintile of grip strength adjusted for sex and BMI^32^. Strength was measured with a Jamar dynamometer, previously calibrated, and the highest value in two consecutive measurements was used in the analyses; and (5) slow walking speed: the lowest cohort-specific quintile in the three-meter walking speed test, adjusted for sex and height^33^.

Based on the aforesaid cross-tabulated bounds of %BF and SMI, sarcopenic obesity was diagnosed when participants were in the upper two quintiles of %BF and in the lower two quintiles of SMI^29^. Likewise, frail obesity was diagnosed when participants were in the upper two quintiles of %BF and met ≥3 Fried criteria.

### Socioeconomic status

Father’s occupation, educational level, former own occupation (almost all participants were currently retired), and current housing conditions were considered as proxies for SES, corresponding respectively to their SES in different periods in life: childhood, youth, adulthood, and late life. Father’s occupation and formal own occupation (corresponding to the current or last job held) were classified according to the National Classification of Occupations in Spain, and grouped into manual and non-manual jobs. Housewives were assigned the occupation of their husbands. Educational level was assessed as the highest level reached (primary or lower, secondary, and university). Individuals were identified as having housing disadvantage when they lived in a house with at least one of the following poor conditions: no elevator in a walk-up building, feeling cold often, or having no heating. Having housing disadvantage was considered as a proxy for current low SES. Having at least one social disadvantage throughout life was considered when they met at least one of the following conditions: father’s manual occupation, primary studies or lower, former own manual occupation, and living in poor housing conditions. A scale was constructed to assess the accumulation of social disadvantages throughout life by adding one point for each previous criteria (range 0–4).

### Other variables

Apart from sex and age, the interview collected participant’s own-reported physician-diagnosed diseases: cardiovascular diseases (heart attack, stroke, heart failure or atrial fibrillation), asthma or chronic bronchitis, osteoarticular diseases (osteoarthritis, rheumatoid arthritis or hip fracture), cancer at any site, and depression requiring drug treatment. Individuals also reported self-rated health and their health-related quality of life (HRQL) using the SF-12 questionnaire from which mental and physical scores were calculated.

### Statistical analysis

The prevalence of sarcopenic and frail obesity was calculated and the chi-square test was used to assess age- and sex-differences. Mean and standard deviation (SD) were used to describe characteristics of the participants in the study according to SES components. We used logistic regression to assess the association of each social disadvantage throughout life with sarcopenic and frail obesity. Regression models were adjusted for age and sex. We used the SES throughout life as a continuous variable to assess the association between the increase in one social disadvantage and sarcopenic obesity or frail obesity. In addition, this scale was divided into three categories (0 or 1, 2, 3 or 4 disadvantages), being 0 or 1 the reference category. A p for linear trend was calculated by modelling the independent variable as continuous. Results are presented as odds ratios (OR) and their 95% confidence interval (CI). We conducted sensitivity analyses with a modified definition of sarcopenic obesity and frail obesity, by considering obesity as BMI ≥ 30 kg/m^2^. Analyses were performed with IBM SPSS for Windows, version 22.0 (Armonk, NY; IBM Corp.), and p < 0.05 was considered statistically significant.

### Data availability

The datasets generated during and/or analyzed during the current study are available from the corresponding author on reasonable request.

## Results

The sample consisted of 1,765 participants, 830 men and 935 women, ranging from 65 to 97 years old. From the total sample, 17.2% (95% CI: 15.4–19.0) had sarcopenic obesity. No differences were found in the prevalence of this condition between men and women, or age groups (Table 1). Sensitivity analysis showed that when BMI criterion was used (≥30 kg/m^2^) instead of %BF to be considered obese, the prevalence for sarcopenic obesity was 5.9% (95% CI: 4.7–7.0). A total of 4.0% (95% CI: 3.1–4.9) of participants had frail obesity. The frequency was higher in women and those aged 75 years-old or above (Table 1). Sensitivity analysis showed that when obesity was defined as BMI ≥ 30 kg/m^2^, the prevalence of frail obesity was similar, 4.1% (95% CI: 3.2–5.0). Both conditions partially overlapped. Among those with sarcopenic obesity, 8.7% were also obese frail. Among obese frail, 36.6% were also obese sarcopenic.

**Table 1:** Frequency of sarcopenic and frail obesity by sex and age, Seniors-ENRICA cohort (2012).

	Sarcopenic obesity			Frail obesity		
	N	n	%	N	n	%
**Overall**	1730	298	17.2	1764	71	4.0
<75	1144	199	17.4	1168	30	2.6
≥75	586	99	16.9	596	41	6.9^a^
**Men**	808	147	18.2	829	12	1.4
<75	549	100	18.2	565	5	0.9
≥75	259	47	18.1	264	7	2.6^a^
**Women**	922	151	16.4	935	59	6.3^b^
<75	595	99	16.6	603	25	4.1
≥75	327	52	15.9	332	34	10.2^a^

Notes: Statistically significant between groups (p < 0.05): ^a^<75 vs. ≥75; ^b^Men vs. Women.

From the total sample, 32.2% (95% CI: 30.0–34.4) had a father with manual occupation, 45.5% (95% CI: 43.2–47.8) had primary or lower educational level, 38.1% (95% CI: 35.9–40.4) had former own manual occupation, and 35.9% (95% CI: 33.6–38.1) had at least one poor housing condition. We noticed a higher frequency of women in all these groups (53.8%, 61.9%, 56.2% and 57.8% respectively). Those in the lowest socioeconomic groups were observed to have worse auto-perceived health, lower HRQL, and greater morbidity. There was a large difference in the frequency of osteoarticular diseases between those with primary or university studies (56.7% vs. 36.0%), and between those with and without housing disadvantage (54.7% vs. 45.6%). Likewise, depression was more frequently reported by those with primary education compared with those with university studies (12.4% vs. 1.5%). No differences were found in the prevalence of sarcopenic obesity across SES categories. However, the prevalence of frail obesity was higher in all the lowest socioeconomic groups. The most pronounced differences occurred between those with primary and university education (5.9 vs. 1.5) (Table 2).

**Table 2:** Sociodemographic and morbidity characteristics of study participants by socioeconomic status components, Seniors-ENRICA cohort (2012).

	Father’s occupation		Educational level			Former own occupation		Poor housing condition	
	Non-manual N = 1196	Manual N = 569	University N = 389	Secondary N = 414	≤ Primary N = 962	Non-manual N = 1092	Manual N = 673	No N = 1132	Yes N = 633
**Sociodemographic**									
Age (years)	72.9 (5.8)	72.3 (6.0)	71.9 (5.6)	71.5 (5.8)	73.6 (5.9)	72.7 (6.0)	72.8 (5.8)	72.8 (6.0)	72.6 (5.8)
Women (%)	52.6	53.8	38.8	45.7	61.9	51.0	56.2	50.3	57.8
**Morbidity**									
Self-perceived health (%)									
Excellent/Very good/Good	66.9	60.3	81.9	75.5	53.0	68.6	58.5	66.8	61.1
Regular/Bad	33.1	39.7	18.1	24.5	47.0	31.4	41.5	33.2	38.9
Physical health score (SF-12)	45.2 (12.0)	43.6 (12.4)	48.0 (10.8)	46.7 (11.2)	42.5 (12.7)	45.6 (11.8)	43.3 (12.7)	44.9 (12.0)	44.4 (12.6)
Mental health score (SF-12)	53.2 (10.6)	52.8 (11.1)	55.2 (8.4)	54.2 (9.0)	51.7 (12.1)	53.5 (10.4)	52.4 (11.4)	53.8 (10.4)	51.8 (11.3)
Cardiovascular disease (%)^a^	6.4	8.3	7.0	7.8	6.7	6.8	7.3	7.2	6.7
Asthma or chronic bronchitis (%)	11.5	12.7	9.8	10.0	13.5	11.9	11.8	11.1	13.3
Osteoarticular diseases (%)^b^	48.3	50.1	36.0	43.2	56.7	46.5	52.7	45.6	54.7
Cancer (%)	3.1	3.5	3.6	2.7	3.3	2.9	3.7	2.6	4.4
Depression (%)	8.4	9.7	1.5	7.5	12.4	7.3	11.3	8.0	10.3
**Sarcopenic obesity (%)**	17.0	17.1	19.1	15.6	16.8	17.2	16.8	18.0	15.2
**Frail obesity (%)**	3.6	4.9	1.5	1.9	5.9	3.0	5.6	3.1	5.7

Continuous variables are expressed as mean ± SD.

^a^Self-reported heart attack, stroke, heart failure or atrial fibrillation.

^b^Self-reported osteoarthritis, rheumatoid arthritis or hip fracture.

Once adjusted for age and sex, none of SES variables were associated with sarcopenic obesity. On the contrary, frail obesity was associated with older age (OR 2.71, 95% CI: 1.67–4.40), being a woman (OR 4.49, 95% CI: 2.39–8.44), lower education (OR 2.66, 95% CI: 1.12–6.32), former own manual occupation (OR 1.83, 95% CI: 1.13–2.98), and having at least one poor housing condition (OR 1.77, 95% CI: 1.09–2.88). Having at least one social disadvantage throughout life also increased the prevalence of having frail obesity (OR 3.45, 95% CI: 1.24–9.61). The likelihood of suffering from frail obesity was increased by 1.49 (95% CI: 1.21–1.85) times for each social disadvantage added (Table 3). Likewise, having 3 or 4 social disadvantages obtained an OR of 3.13 (95% CI: 1.71–5.70) when comparing to 0 or 1 disadvantages (Table 4).

**Table 3:** Association between presenting sarcopenic obesity or frail obesity and sociodemographic characteristics and socioeconomic status, Seniors-ENRICA cohort (2012).

	N	Sarcopenic obesity		N	Frail obesity	
		n	OR (95% CI)^a^		n	OR (95% CI)^a^
**Age**						
<75	1144	199	Ref.	1168	30	Ref.
≥75	586	99	0.97 (0.74–1.26)	596	41	2.71 (1.67–4.40)
**Sex**						
Men	808	147	Ref.	829	12	Ref.
Women	922	151	0.88 (0.67–1.14)	935	59	4.49 (2.39–8.44)
**Father’s occupation**						
Non-manual	1168	201	Ref.	1195	43	Ref.
Manual	562	97	1.00 (0.77–1.31)	569	28	1.38 (0.84–2.26)
**Educational level**						
University	382	74	Ref.	389	6	Ref.
Secondary	406	64	0.78 (0.54–1.13)	414	8	1.17 (0.40–3.43)
≤Primary	942	160	0.89 (0.65–1.21)	961	57	2.66 (1.12–6.32)
**Former own occupation**						
Non-manual	1072	186	Ref.	1091	33	Ref.
Manual	658	112	0.98 (0.76–1.27)	673	38	1.83 (1.13–2.98)
**At least one poor housing condition**						
No	1110	203	Ref.	1131	35	Ref.
Yes	620	95	0.81 (0.62–1.06)	633	36	1.77 (1.09–2.88)
**At least one social disadvantage throughout life**						
No	352	63	Ref.	358	4	Ref.
Yes	1378	235	0.96 (0.71–1.31)	1406	67	3.45 (1.24–9.61)
**The increase of one social disadvantage**	1730	—	0.96 (0.87–1.07)	1764	—	1.49 (1.21–1.85)

^a^Model adjusted for age (as a continuous variable) and sex (except for the stratification variable).

**Table 4:** Association between the number of social disadvantages throughout life and the probability of suffering from sarcopenic obesity or frail obesity, Seniors-ENRICA cohort (2012).

	Number of social disadvantages throughout life			
	0/1	2	3/4	p-trend
**Sarcopenic obesity**	153/850	72/458	73/422	
OR (95% CI)^a^	Ref.	0.86 (0.63–1.17)	0.97 (0.71–1.32)	0.726
**Frail obesity**	18/868	21/465	32/431	
OR (95% CI)^a^	Ref.	1.89 (0.98–3.61)	3.13 (1.71–5.70)	<0.001

Notes: OR = Odds ratio; IC = Confidence interval.

^a^Model adjusted for age (as a continuous variable) and sex.

## Discussion

This cross-sectional analysis conducted with a population-based study in Spain, showed a prevalence of sarcopenic obesity of 17.2%, and a prevalence of frail obesity of 4.0%. No association was found between SES and sarcopenic obesity. In contrast, lower educational level, having worked in manual jobs, and having poor housing conditions were associated with frail obesity. In addition, having at least one social disadvantage throughout life substantially increased the prevalence of having frail obesity.

Few studies have determined the prevalence of sarcopenic obesity in Spain, reporting a slightly lower frequency (14.9% vs. 17.2%), also using sex specific quintiles of %BF and SMI^14^. In Europe, a study performed between 2011 and 2012 showed, by means of predictive equations, that 11.0% of the 1,865 Spanish participants aged ≥65 had sarcopenic obesity^13^. In France, a lower prevalence was also found from the analysis of 1,308 institutionalized healthy women aged ≥75. Of them, 2.8% were identified as obese sarcopenic^34^. In the US, a cross-sectional analysis performed with 2,287 subjects aged 60 and older from the National Health and Nutrition Examination Survey (NHANES 1999–2004) reported that 10.4% were obese sarcopenic. In this case, waist circumference was used to assess obesity, and appendicular SMM was measured by dual-energy X-ray absorptiometry (DXA)^35^. Finally, data from the Nutrition as a Determinant of Successful Aging (NuAge) Study in Canada, with 904 community-dwelling individuals aged between 68 and 82 showed a prevalence of 18.8% in men, and 10.8% in women (the European Working Group on Sarcopenic in Older People criteria were used for sarcopenia diagnosis, and body composition was assessed by DXA)^36^.

Marked differences exist in the prevalence among studies. Comparison of results on sarcopenic obesity is still somewhat complex due to the heterogeneity of the methods and cut-off points used to assess both SMM and obesity. Since first defined sarcopenia in 1998 by Baumgartner *et al*. as appendicular SMM (kg/m^2^) less than two standard deviations below the sex-specific mean in a young reference group^37^, several definitions have been proposed relying on muscle mass, but also on strength and physical performance^38^. Coupled with this problem, the lack of consensus in determining high fat mass makes sarcopenic obesity prevalence vary widely^39^. In fact, in our analysis it ranged from 17.2% using %BF to 5.8% using BMI as diagnostic criteria. However, prevalence of frail obesity was similar when using BMI ≥ 30 kg/m2. The use of BMI as an indicator of obesity in the elderly has been questioned due to changes in body composition with ageing, since it does not take into account the loss of muscle mass^40^. On the other hand, the utilization of computed tomography and magnetic resonance (considered as the gold standard methods in research), is of limited use in epidemiological studies due to the emission of radiation and high cost, being DXA and BIA (considered to be a portable alternative to DXA) the most common instruments to assess sarcopenic obesity in general populations.

Influence of SES on health has been widely reported. It is known that, social inequalities begin in childhood, and can be sustained throughout life, but the way they impact negatively on some health events later in life remains unclear. Educational level, as a SES indicator in youth, constitutes a strong predictor of future type of employment, and it is considered the socioeconomic factor with most influence on health. Thus, educational inequalities have been related with a worse HRQL in the elderly^41^. Likewise, studies conducted both in Europe and USA, have reported an increased risk of mortality in those with lower educational level^21,22^. Among middle age participants, manual work could involve greater exposure to certain conditions, which may influence physical and mental health in the elderly^18,19^, as well as in achieving a better HRQL after labour market^42^. Midlife adversities like low occupational position or high job strain have been associated with post-retirement depressive symptoms^18^. Besides, higher midlife occupational physical activity levels, have been related to major risk of disability later in life^19^. Finally, it is probable that all previously mentioned factors could influence people to have less access to a better equipped house later in life. There are grounds for believing that older adults are probably more exposed to certain social disadvantage like lower purchasing power. It is also known that, retired people are at greater risk of physical disability and frailty^43^, and those conditions increase in those with fewer resources^20^.

Some information on SES and its relationship with sarcopenia and frailty is available^24,25^. Though, data studying this association with sarcopenic obesity is scarce^26^, whereas no study has evaluated its link with frail obesity. Moreover, SES throughout life has never been assessed. We observed no association between sarcopenic obesity and any of the SES variables. These data are in line with those reported by Tyrovolas *et al*. who found similar results when assessing SES as educational level and wealth^26^. Our results showed an association between being in social disadvantage and suffering from frail obesity. Interestingly, all the SES determinants were positively and statistically associated with frail obesity, except father’s occupation, that also is the furthest in the causal pathway. Moreover, those with at least one social disadvantage throughout life presented a probability up to threefold higher risk of being obese frail than those without any social disadvantage.

We observed that sarcopenic obesity appears more frequently among those with higher levels of education, while frail obesity does among those with just primary studies. It is likely that more educated subjects may have lower muscle mass, probably because they have more sedentary jobs. However, it is plausible that their SES allowed them to access better health services, and associates better nutritional habits and self-care which may have avoided the frailty characteristic loss of function. This reason justifies that education, as a determinant of health, should be part of an integral form in the new approach to public health.

To the best of our knowledge, this is the first study to evaluate the association between frail obesity and SES determinants in community-dwelling older adults. It has the strength of having been carried out by means of standardized protocols. Additionally, a basic adjustment was performed. However, it has also several limitations. The main one is the cross-sectional nature of the data that may suffer from survival, recall, and selection biases. Owing to the difficulty obtaining self-reported information on income among the elderly, we did not consider this variable, even though, the best way to measure SES is probably combining education, occupation, and income. In addition, a small number of cases in some stratified groups were found, especially for frailty. Also, the use of specific quintiles in our sample to define the events makes it difficult to generalize our results, due to the lack of normative cut-off points.

## Conclusions

This study emphasizes the need for consensual criteria in the diagnosis of these syndromes, and to point out the need for an agreement on the definitions, cut-off points, and measurement methods, in order to be used in general populations of older adults. At the same time, this research highlights the special importance of education and SES throughout life in the prevalence of frailty related to obesity, but not for sarcopenic obesity. This probably implies that for frail obesity, determinants could have been operating for a long period of time, implying a complex process beginning early in life.
